# Childhood onset exercise addiction or atypical anorexia nervosa during Covid-19: case report

**DOI:** 10.1186/s40337-021-00450-4

**Published:** 2021-08-09

**Authors:** F. McNicholas

**Affiliations:** 1 Department of child & adolescent psychiatry, School of Medicine and Medical Sciences, Belfield, UCD, Dublin 4, Ireland; 2 Our Lady’s Children’s Hospital, Crumlin, Dublin 12, Ireland; 3 Lucena Clinic Rathgar, Dublin 6, Ireland

**Keywords:** Childhood-onset, Anorexia nervosa, AN, Exercise addiction, Covid-19

## Abstract

**Background:**

Childhood-onset Anorexia Nervosa (AN) is recognised to be atypical in presentation, both in terms of extent and nature of eating pathology, exercise and compensatory behaviours with many falling short of full diagnostic criteria. Failure to consider an eating disorder diagnosis in youth who present with extreme weight loss may have serious immediate and long term implications. However, failure to consider other non-organic causes of weight loss may be equally detrimental to the child’s health.

**Case presentation:**

This case reports on the acute presentation of a 12-year old boy, who presented to hospital in a severely malnourished state eight weeks into lockdown. To compensate for Covid-19 induced restrictions on sporting activity, this boy had followed a self-imposed daily schedule of arduous exercise, without increasing his nutritional intake. This report examines the clinical features suggestive of AN and other differential diagnosis. A discussion on the specific diagnostic differential of exercise addiction and challenges faced by youth during Covid-19 restrictions are presented.

**Conclusion:**

Accepting that AN may present atypically in pre-pubertal youth, it is important that clinicians maintain an open mind in youth presenting without goal directed weight loss. Although weight loss was significant in this case, it was due to an excessive exercise regime. This may have commenced as a coping strategy in response to Covid-19 restrictions but subsequently became excessive and impairing in nature. The collateral damage of Covid-19 mandated restrictions, aimed at containing the spread of the virus, are evident in this case. Clinicians need to be alert to potentially maladaptive coping strategies and unusual or altered pathways of presentation, especially in younger children during these challenging times.

## Background

Anorexia Nervosa (AN) is a disorder characterised by self-imposed food restriction resulting in weight loss coupled with an intense fear of weight gain, or of being fat, and an over-evaluation of body weight and shape (DSM5, 2013). Associated behaviours may include over-exercising, binge eating, purging and use of diet pills or laxatives. In children failure to progress along expected weight trajectories occurs with detrimental effects on physical health. Although AN typically has its age of onset in adolescence, it may present both in pre-pubertal children and older adults. Presentation in younger children may be atypical with proportionally more boys presenting; ratio of girls: boys 4:1 as opposed to the adult ratio of 10:1 [1] and 5:1 in United Kingdom and Ireland [2].

Of particular concern is that prevalence rates of AN have more than doubled in recent years (Steinhausen & Jensen [3], increasing both in adolescents and younger aged children [2]. Rates of hospital admissions have also increased [4]. The most recent community-based studies using DSM-5 criteria reported estimated lifetime prevalence of AN of 6.2% for females and 0.3% for males [5]. Poorer prognostic outcomes are a concern in pre-pubertal youth. AN is recognized to have high morbidity and mortality rates [6]. In a large study (*n* = 68) of hospital treated youth under 14 diagnosed with AN, followed up for a mean of 7.5 years, only 41% were reported as having a good outcome, defined as achieving a normal body weight, and if applicable, return of regular menses [7]. Given low body fat stores in children, weight loss rapidly leads to medical compromise and early onset AN more often presents to the paediatrician and results in hospital admission [7]. Early recognition and appropriate treatment is essential to optimise treatment outcomes. The aetiology of AN is not fully understood and treatment often assumes an agnostic approach. Its origins are considered to be multifactorial with contributions from cultural, familial, individual, genetic and biochemical domains. More recent candidate gene meta-analyses suggest a putative link with serotonin genes [8], whilst evidence supporting the neurobiological underpinnings are supported by advances in neuroimaging research, using structural and functional neuroimaging research [9, 10]. Adverse childhood events, stressors, bully-victim status and personality traits of perfectionism have also been suggested [11].

Following the declaration of the Corona Virus infection as a pandemic by the WHO in March 2020, governments around the world instigated various measures to contain the virus. In Ireland, as with many countries, these included closure of all non-essential businesses, schools and universities, restriction were imposed on gyms, sporting and training events and limits were placed on social gatherings. Many of these have had a direct impact on the lives of children. Family life has also been disrupted for many, with temporary and permanent job losses, working from home orders, and for some, combining work with home schooling. Data is emerging of adverse psychological effects in adults [12] and in children resulting from Covid-19 pandemic [13]. Given the salience of social contact and peer relationships during childhood, it is understandable how school closures and reduced social contact have been associated with increased loneliness, anxiety and low mood in youth during the pandemic [14]. Specifically, limitations on social gatherings and movement have been found to be associated with reduced levels of physical activity, higher sedentary behaviours and more screen time among Canadian youth aged 5–17 surveyed [15]. Large scale population surveys among adults have also shown significant effects on eating and exercise behaviours, [16]. A robust systematic review concluded that home confinement linked to COVID-19 was adversely affecting people’s lifestyles with a tendency towards poorer food choices, over eating and increased weight [17]. A smaller number of studies have reported on changes in eating and dietary habits among youth. In a cross sectional study of Egyptian youth, 37.4% reported an increase in weight with an increased prevalence of unhealthy food consumption (24.2%), night eating (29.3%) and emotional eating (17.9%) [18]. Overeating was also prominent in a large cohort (*n* = 2996) of school aged youth (mean age 15.4) in Italy, and attributed to ‘feeling bored’ (present in 35.8% sample) and ‘having nothing else to do’ (19.7%) [19].

In a national survey relating to post-pandemic changes in eating and exercise behaviours in Australia, higher levels of restrictive and maladaptive eating behaviours were seen compared to the general population [16]. A systematic literature review conducted by Mehta [20] including cross sectional and longitudinal studies, concluded the majority of individuals with pre-existing eating disorders reported an increase in anorexic symptomatology. This was also the perception for 41% of health care providers) for adults with AN, [21].

Clinical referrals of cases with eating disorders among adults and youth have also increased exponentially over this time, being referred to as a ‘outbreak of Anorexia Nervosa admissions’ by one group due to an observed 104% increase [22].

Increased weight-control behaviours seen have been attributed by some authors to reflect a defence against Covid-19 enforced loss of control [23], or as a consequence to boredom and loss of structure [19]. Reports have also described increased care-giver burden experienced by carers during lockdown, along with the urgent need to maintain but modify access to care [24]. Qualitative reports have offered insights into the difficulties brought about by these restrictions, but such reports have generally been restricted to adult cohorts [25, 26].

Interestingly, some studies have highlighted health promotion changes during lock down, with improved self-reported exercise in almost half of one sample studied [16]. Increased exercise was viewed as a positive coping strategy for 40% of youth with stable weight status (42.5%), lack of emotional eating (56.2%) and increased consumption of healthy foods (25.1%) [18]. Adverse effects of exercise, unrelated to prior eating concerns, have to date not been described.

This paper reports on the acute presentation of a young boy, with no prior parental concerns regarding his eating, who presented to hospital in a severely malnourished state eight weeks into lockdown. It examines clinical features suggestive of AN and other eating disorders, it considers other differential diagnoses, and explores treatment implications. It examines the impact of Covid-19 linked restrictions on social gatherings and physical activity on his presentation. Ethical exemption was granted and written consent provided by the family. The initials AB have been assigned and minor details changed to protect subject anonymity.

## Case presentation

### Presenting complaint

AB is a 12 year old boy admitted to the paediatric hospital following precipitous weight loss as a result of increased exercise, in an otherwise very active and sporty boy. According to his mother, this commenced the ‘day after lockdown’ and was not linked with any intentional reduction in food intake or body image dissatisfaction. In fact AB maintained the position that *he was too thin*. AB joined the family for meals as per norm with no reduction in usual amounts or variety consumed and there was no obvious change in his mood state.

During Covid-19 restrictions, AB missed attending school, socialising with peers and his training routine. He recreated this with a self-initiated exercise schedule. At the time of lockdown, AB’s father had been abroad and his absence from the family home was further prolonged by a two-week enforced post-holiday quarantine. AB’s mother was balancing a heavy work-at-home schedule with providing for her other children and the extent of AB’s exercise and associated weight loss, in the presence of usual eating and general demeanour had gone unnoticed until father’s return. It was at that point that AB’s mother attended her family physician, and was advised to seek hospital admission due to significant weight loss.

### Nutritional history

AB was described by his mother as pre-morbidly being a ‘faddy eater’, showing little interest in food and eating much less than his younger siblings, but without any adverse effects. An example given was during their pre-Covid ski holiday, all the family increased their food intake apart from AB. Although AB ate fast food when out with friends, at home he preferred simple, minimally flavoured, bland foods and rarely ate treats. For ease of meal preparation, AB’s mother had a scheduled weekly meal plan which all the family, including AB, followed. He never expressed any concern over his body image and there were never any deviations from his growth trajectory. Mother estimated his typical daily intake to be 1300–1500 cal/day.

### Exercise history

AB was talented athletically, played soccer and football competitively, with a heavy but structured schedule of daily soccer training and weekend competitive games with two different clubs. He also spent time on the family trampoline at home. This amounted to 6 h strenuous exercise per week, plus routine school sports. In the days immediately after lockdown, to replace his previously structured day, AB started running for up to an hour a day, covering 10-11 k, initially alongside his mother’s daily walk, and later independently and much faster. He later joined his brother in cycling 10-20 k daily and spent 1–2 h each evening on the family trampoline, irrespective of weather. The estimated daily calories expenditure was around 2600 cal/day, and greater than the reference range of 1800–2200 for an active 9–13 year old boy.

### Weight history

Pre-morbidly AB’s weight was estimated at 33.1 kg, corresponding to a Body Mass Index (BMI) of 15.6 (15th %) or 90% Ideal Body Weight (IBW). He had lost almost 5 k in 2 months and was medically comprised on admission. His admission weight was 27.5Kg, BMI 12.9 (0.03%) corresponding IBW 70.35%. At the time of admission and based on maternal report and discussion with the hospital dietician, AB’s daily calorie intake was estimated to be between 1300 and 1500 Calories/day, giving an overall deficit of 1100–1300 cal/day. (A sample daily intake in included in Table 1.)

**Table 1:** Sample Meal plans pre admission and post discharge

	Breakfast	Snack	Lunch	Snack	Dinner	Snack	Total calorie estimate/day
Pre-admission	Krispies, full fat milk, apple juice 400 cal	Juice Grapes 100 cal	Brown bread butter chicken Water 400 cal		Salmon with chilli and lemongrass, couscous, olive oil, lettuce and sweetcorn Juice 450 cal	1–2 digestive Biscuit Water 140 cal	1490 cal
Discharge	Krispies, full fat milk, apple juice 400 cal	3 digestive Biscuits and banana 320 cal	Brown bread butter, 2 chicken goujons Juice 450 cal	2 crackers, butter, cheddar, grapes Milk 400 cal	Homemade Thai chicken green curry, rice, 2 dough balls 400 cal	3 digestive Biscuits and 16 small grapes 260 cal	2230

### Developmental history

AB’s developmental milestones are within normal boundaries. He was perceived to be popular at school with many friends and academically very able. He engaged well with his family, was generally very active but not considered to have difficulties with attention or impulsivity. There was no suggestion of any pre-morbid anxiety, obsessional features or low mood. Although he generally liked routines, schedules and well planned activities, his mother was adamant that there were no social communication difficulties. He was self-sufficient, empathetic and not overly emotional, tending to deal with difficulties or upsets himself. Given his mother’s work with children with special needs, she felt that this was a precise and accurate reflection of his development. Teacher reports described a biddable boy, who excelled in school and sports, with a close circle of friends. He was described as helpful and considerate. There were no concerns expressed by teachers regarding mood, social communication difficulties, or ADHD. There was no prior contact with mental health services and no history of substance misuse. There was no medical history of note, he was up to date with all his vaccinations, had no allergies and was not on any medication.

### Family history

AB is the middle of 5 healthy children aged from 17 to 10. Mother describes some personal difficulties with weight maintenance and past dieting behaviour, with weight fluctuation of 2 stone. At the time of presentation, she was happy with her weight and had a structured exercise routine built into her day to facilitate weight maintenance. Father, like his siblings, is tall and thin. All AB’s family are sporty, of slim physique and his older brother was a national athlete for many years. Mother described the family to follow a ‘healthy diet of home based and natural ingredients’ by which she meant that she prepared fresh meals daily using fresh produce and limited her use of processed foods (Table 1). She also reported she limited her childrens’ use of social media. AB parents describe a happy marriage and no difficulties with co-parenting. Parents described an authoritative parenting style, with low levels of expressed emotions and a preference for advanced planning, routine and structure. By way of example, holidays aboard were planned well ahead of time including scheduling and booking activities on various days. There were no other family stressors reported. There was a history of bipolar disorder on the paternal side, and depression in maternal first degree relative.

### Sociocultural factors

AB was pre-pubertal, with no expressed concern regarding male gender. He was perceived favourably by peers and had not experienced any bullying at school. Although the clinicians felt father’s absence and forced quarantine during the immediate lockdown might have triggered additional anxiety for AB and his family, and contributed to his presentation, this was disputed by both AB and his mother.

### Mental state examination

AB was extremely thin, gaunt with a very visible skeletal frame. He found it hard to engage and eye contact was generally poor. Speech was low in volume and conversation restricted. AB described his mood as mostly ‘sad’ and ‘bored’, he reported difficulty adjusting to lockdown, he missed his friends and soccer training and found the days at home long and uneventful. He reported a preference to have ‘things planned’ and felt better when he joined his mother or brother on their activities. He denied his pursuit of exercise was driven by any wish to alter his body shape or to lose weight. He acknowledged he was underweight and stated he ‘did not like being this skinny’. He perceived that his engagement in exercise was ‘80% to stop being bored and 20% to keep fit’ and felt it aligned with the behaviour of his other family members. He recognised that he had become ‘obsessed’ with a desire to exercise and its mood elevating component. If he did not exercise for any reason, which he reported was very seldom, he reported feeling ‘sad’. He volunteered that immediately pre- admission he exercised less because he was too tired. AB denied any fear of an untoward outcome if he did not exercise as planned and allowed his brother to choose their cycle route, distance and timing. Although he chose his exact running route, his start time was linked to that of his mother’s walk. During this time, he denied any attempt to limit his calorie intake and stated he enjoyed his meals. He denied any feelings of hunger; ‘it had never occurred to me to eat more, and no one told me’. He described his mood as good unless he was unable to exercise for any reason and denied any ideas or behaviours linked to self-harm. There was no evidence of any abnormal thought form or perceptual abnormalities. His thought content was very much focussed on his desire to return to his routine of school, sports and time with friends. He was eager to follow the hospital treatment plan and be discharged.

### Medical examination

Physical examination revealed a cachectic boy, with low body temperature (35.8–36, Normal 37.) There was evidence of cardiac insufficiency; low heart rate (30s at night time) and orthostatic changes of 22 bpm (lying HR 38/ standing HR 60). His blood pressure was variable; systolic ranging from 81 to 105 mmHg, diastolic from 52 to 76 mmHg, but with minimum orthostatic changes (< 10 mmHg). His electro-cardiograph revealed sinus bradycardia with normal QTc. There were some initial abnormalities in his biochemistry and haematology results (Table 2).

**Table 2:** Blood tests

WCC	RBC	U&Es	Liver Function
2.8 (4.5–13.5)	Plat 140 (150–450) RBC 3.72 (4.2–5.2) Hg 107 (115–155)	Na130 (135–145) K 2.9 (3.6–5)	Protein 59 (60–80) AST 42 (< 40) ALT 53 (< 35) LDH 788 (233–600) Urea 7.3–8.3 (2–6)
Phosphate 47 50–350)	Inorganic Phos .61 (1.2–2)	Mg 0.68 (0.7–1.1)	Ca 2.38 (2.4–4.33)
TFT Normal	Parathyroid Normal.		

Normal laboratory reference ranges are in parentheses

### Psychometric scales used

Child Behaviour Check List (CBCL) completed by parents suggested no areas of clinical concern. AB completed the Rosenberg Self-Esteem scale, a self-esteem measure widely used in social-science research and helpful to examine self-esteem. Scores below 15 indicate problematic low self-esteem [27]. AB scored 40/40. The exercise addiction scale was also completed. This is a short screening tool used clinically to examine the possibility of exercise addiction, with scores above 24 being considered clinically relevant. AB scored 29/30, indicating significant difficulties. By contrast his global score on the Eating Disorder Examination Questionnaire (EDE-Q) was 0.39, with very low scores on each of the subscales: Restrain: 0; Shape: 0.75 and Weight 0.8, suggesting no eating disordered pathology.

#### Impression

At the time of admission AB was severely undernourished, having lost an excessive amount of body weight in a short time. This was due to a significant imbalance between energy expenditure and intake but without any evidence of eating disordered psychopathology. Specifically, AB did not endorse a fear of fatness or weight gain, body image dissatisfaction, or a distorted view of body shape. His excessive engagement in exercise was driven by a desire to impose structure on his day and fight boredom. It was subsequently reinforced by an improvement in his mood. He did not meet criteria for AN or Atypical AN, included in Other Specified Feeding or Eating Disorders (OSFED). A working diagnosis of exercise addition was made (Table 3).

**Table 3:** Diagnostic Criteria for AN: DSM-5 compared with AB’s presentation

Diagnostic Features of AN	AB
Significant food restriction leading to failure to continue along developmentally appropriate weight trajectory	No food restriction; calories consumed were as before but inadequate for energy expended
Fear of becoming fat or gaining weight	No fear of weight gain, rather a recognition of being ‘too thin’ and a willingness to regain weight lost
Over valuation or distorted view of body weight and shape (or parts of body)	Perceives self as underweight, eager to return to pre-morbid state No desire to alter body shape by exercise
Behaviour motivated by desire to avoid weight gain, maintain low body weight	Exercise was driven by a desire for structure and became positively re-enforced by mood boosting effect

### Progress on admission

AB treatment plan was devised following multi-professional input from child psychiatry, paediatrics and dietetics. He commenced on a refeeding program, with a gradual increase from 1400 to 2000-2400 cal/day during hospital stay with phosphate and thiamine supplements. He found it very difficult to eat all the food offered, initially eating as little as 400–500 cal/ day. This low intake was driven by severe abdominal discomfort, reflux and severe constipation upon refeeding. Replacement with a nutritional supplement, *Fortisip,* was given. AB had no bowel movements over a 4 week period despite heavy doses of laxatives. Clinical examinations and plain film of abdomen did not reveal any evidence of impaction. His mood dropped significantly as he struggled to adhere to his meal plan, tolerate painful abdominal peristaltic movements and gain the necessary weight needed for discharge. One-one nursing was provided at meal times to support AB with oral intake, ensure postprandial bed rest and observe if any desire to exercise. His parents also struggled with what they perceived to be the multi-disciplinary team’s over focus on weight restoration and a fear that AB’s complaints were misinterpreted as wilful refusal, rather than an inability to eat. They considered discharge against medical advice.

An early intensive transitional out-patient plan was progressed to facilitate family engagement and assist with careful weight restoration. This followed an adapted version of Family based treatment, with psycho-education offered, provision of structured meal plans, and establishing parental role in all aspects of food preparation, presentation and supervision. AB was allowed trials home for family meals and over-nights, despite being medically compromised, and these were carefully monitoring by his mother and clinical team. A detailed record of all food offered and eaten was kept and reviewed at hospital reviews, along with estimated daily calories (circa 2100 cal/day). Initial progress was followed by a significant drop in weight and low sodium which precipitated a medical re-admission and a need for cardiac monitoring. AB admitted he had been spitting out half of the food plated by his mother for fear of a return of his abdominal pain.

After one week of medical stabilization as an in-patient, transitional care continued with twice weekly psychiatry/medical review and heightened maternal supervision. AB was discharged to community child and adolescent mental health services (CAMHS) after 2 weeks. His weight at discharge was 30.4 kg, BMI 14.5 and IBW 82.6%, still below his pre-morbid levels. Zoom out-patient sessions were planned with CAMHS given the reduced face-to-face contact during Covid-19. AB found these sessions very difficult, finding it hard to engage and missing out on non-verbal cues. Subsequent Zoom calls continued with his mother who reported on weekly weights and the degree of adherence to the meal plan. This allowed daily calorie intake to be estimated and he was eating 1800–2000 cal per day without resistance. AB’s mother also reported on the return of any physical symptoms and the degree of AB’s re-engagement with family and social life. Casual sporting activities were gradually re-introduced, alongside additional snacks. With time, and restoration of initial weight lost, additional snacks were dictated by preference rather than imposed. AB was discharged from CAMHS eight weeks post-hospital discharge. Two months post-discharge and 6 months post- initial presentation, AB’s mother wrote a letter updating the clinical team as to AB’s ongoing progress. She enclosed a photo of AB enjoying a ‘McDonalds’ equivalent. She reported he was ‘back to his normal self’ with resumption of pre-morbid eating habits and reaching his pre-morbid weight. AB did not receive any neuroleptic medication during admission, and prescription of thiamine and laxatives had been discontinued.

### Covid-19 impact

AB had created a daily routine immediately following the imposed lock down and loss of his previously busy schedule of football training and competitive matches. Initially his pursuit of physical activity followed the family’s engagement in health optimisation during Covid-19 and was pursued as a shared activity. Within a few weeks it surpassed it and seemed to take primacy over other activities. AB reported being increasing driven to, and rewarded by, the mood boosting effects of his exercise, and being unaware of any hunger sensations, continued with his previous scheduled meal and snack routines. He maintained contact with some friends through social media, but had not socialised with any face to face. The family coped as best they could with the additional stressors of parents and children working and studying from home. The delay in AB’s presentation was most likely due to mother having to manage on her own while her husband was in quarantine. The reduced ability of face-to-face clinical sessions made engagement with ongoing mental health services difficult for AB, due to his difficulty picking up subtle non-verbal cues and his difficulty with emotional intelligence. A decision to work flexibly and independently with his mother, using principles from FBT approach, supporting and empowering her to monitor AB’s nutritional intake and physical state, allowed safe medical monitoring.

## Discussion and conclusions

Childhood-onset Eating Disorders (ED) describes the onset of eating psychopathology in children under the age of 13 years. Although the DSM-5 does not have any specifiers for childhood onset AN, it is recognised in the literature to present in an atypical fashion, both in terms of degree and type of eating pathology, exercise and compensatory behaviours, with many falling short of the diagnostic criteria for AN [1, 2].

Although early onset cases present with lower self-reported eating psychopathology, lower rates of binging, purging and less engagement in excessive exercise as a means of weight and shape control, early onset cases have been reported to lose weight faster, presenting with lower percentage of ideal body weight on admission [28]. Rapid weight loss in children places them at high risk of medical destabilisation given they have less adipose tissue than older aged youth [1]. Studies that have examined outcomes have also highlighted family and healthcare burden, with two-thirds of cases still in active treatment one year later.

Given the atypical presentation described in the literature, especially the varied endorsement of typical eating psychopathology, and proportionally higher rates of presentation in boys, such youth may be at risk of delayed diagnosis and treatment. They may also be at risk of iatrogenic harm by nature of inappropriate investigations for weight loss. The alternative is also true; an assumption that all weight loss, or a high drive to exercise, relates to an undisclosed eating disorder might also lead to inappropriate treatment, therapeutic fatigue and family disengagement. Although AB never endorsed any eating psychopathology during his stay and he showed a willingness to eat high calorie foods to speed up his recovery, his parents felt that some of the clinicians were working on the assumption of an undisclosed, or yet to emerge, eating disorder. This led to difficult family-clinician engagement at times, including a discharge against medical advice event. The steady, if slow, progress, the remittance of AB’s gastric symptoms and the ongoing commitment by his parents to adhere to a mutually agreed safety plan all allowed an opportunity to continue working therapeutically with the family and consider alternative diagnosis.

At the time of admission AB was severely undernourished having lost an excessive amount of body weight in a short time. This was secondary to a significant imbalance between energy expenditure and food intake. AB’s increase in physical activity post lockdown was motivated by a desire to impose a routine and re-enforced by mood enhancement. It exceeded his premorbid activity levels, whilst his food intake and eating schedule remained at pre-pandemic levels. His failure to increase his nutritional intake commensurate with energy expenditure was due to a lack of hunger and general disinterest in food, rather than intentional food restriction. AB did not endorse a fear of fatness or weight gain, body image dissatisfaction or a distorted view of body shape. He did not meet criteria for AN. Equally AB’s presentation was not consistent with Atypical AN, included under Other Specified Feeding or Eating Disorders (OSFED) in DSM 5, and previously known as Eating Disorder Not Otherwise Specified (EDNOS). Such a diagnosis is considered when prominent eating disorder symptoms are present, including a restricted eating pattern, but the person’s weight remains within normal range. At the time of presentation, AB was underweight and without specific eating psychopathology. Cross sectional diagnoses may change and be revised over time making AN (and ARFID discussed below) likely differential diagnoses.

Avoidant/restrictive food intake disorder (ARFID) describes a condition where there is a persistent failure to meet appropriate nutritional or energy requirements such that intervention is required. The food avoidance or refusal may result from a feared aversive consequence from eating, food avoidance based on sensory characteristics of food such as taste, smell or consistency, or a lack of interest in eating. Typical eating psychopathology as seen in AN, such as body dissatisfaction, fear of weight gain or drive for thinness, are lacking. For a diagnosis of ARFID to be made, the consequences of food restriction must be associated with one of the following; significant weight loss, faltering growth or nutritional deficiency, dependence on nasogastric feeding or nutritional supplements, or a significant impact on psychosocial functioning. A diagnosis of ARFID was considered, in that AB presented with low weight, was in need of medical management and nutritional supplements, and there was evidence of adverse medical and psychosocial functioning. However, the salient feature of food restriction causing the weight loss was not present and although AB had a rather limited range of foods eaten, with strong preferences for routine, he was able to deviate from this if required, for example in social situations. Additionally prior to his hospital admission, his restricted eating habits had not led to any deviations from his growth trajectories. Although AB had shown a lower level of interest in foods than his siblings, this pre-dated his weight loss, and had not changed over this period. AB’s weight loss was as a result of increased energy expenditure and failure to increase his food intake to negate this nutritional deficit.

The clinical presentation also did not support a diagnosis of Obsessive Compulsive disorder (OCD), characterised by intrusive and unwanted obsessional thoughts, images or urges. AB’s engagement in exercise was deliberate, self-driven and enjoyable. In OCD, the associated repetitive behaviours or compulsions are often aimed at reducing the associated anxiety. Whilst AB was regretful when unable to carry out his exercise routines as planned, there were no feared consequences or escalation of anxiety. In fact, AB did not present with any anxiety symptoms, the only negative mood state being one of sadness due to Covid-19 related restrictions, and subsequent hospital admission.

A diagnosis of autism spectrum disorder was also considered, but not substantiated. The developmental history offered by his mother did not suggest difficulties with reciprocal friendships, empathy, or social-emotional reciprocity. There was no report of restrictive or repetitive behaviours or interests. Whilst there may have been reduced expression of nonverbal communication, evidenced at time of assessment, it was present at home and more likely to have been influenced by his negative mood state commensurate with admission. Both at home and in the hospital, AB used language to communicate feelings (albeit with some reluctance) and engaged in reciprocal conversation. Furthermore, there were no concerns expressed by teachers in this regard. He was perceived to be pro-social and popular.

### Exercise addiction

A working diagnosis of exercise addition was considered most appropriate (Table 2).

Exercise is generally considered as healthy and mood enhancing and was recommended as a way to stay healthy during the pandemic. However, unrelenting pursuit and obsessional engagement in exercise, to the point of injury, over-use or disengagement from other activities, is problematic. Prevalence studies range from 0.5 to 52%, depending on whether studies are conducted in the general population (0.5%) or higher risk groups, such as competing triathletes [29]. There are no available studies on prevalence rates in children. Exercise addiction shares many of the constructs more typically associated with addictive behaviours, such as the salience or importance of the activity, sense of loss of control, need to engage in increasing amounts and the experience of withdrawal symptoms when thwarted. The mood enhancing effect may also drive behaviour. Many of these features applied to AB; his exercise regime increased over the weeks and he opted to pursue them ahead of social engagement with family or alternative activities. He was also able to acknowledge the mood boosting components, pre-exercise anticipation and enhanced mood following his daily schedule. When disallowed following hospital admission, he felt irritated by his immobility but was also somewhat fatigued by his weakened medical state.

The Exercise Addiction Inventory (EAI) offers a structured and reliable method of self-report [30]. It is a short screening tool with good psychometric properties. Using six general components of addiction, responses are given along a 5-point Likert scale with a range of 6–30 points; scores greater than 24 reflective of exercise addiction. Whilst pursuit of exercise is often part of an eating disorder presentation, excessive exercise may occur in the absence of any eating psychopathology and in the absence of any reduced health-related quality of life [31]. Personality traits such as perfectionism and narcissism, often associated with ED, have been described. These traits are considered to contribute to athletic excellence and an ability to withstand high degrees of bodily distress in terms of pain and exercise related injuries [31]. However excessive exercise, not met with adequate nutrition, can lead to serious weight loss, body disfigurement and illness, along with loss of insight. Treatment is directed towards re-establishing health and moderating the level of activity, through cognitive behavioural approaches. Medication may be helpful for any associated depression, anxiety or medical compromise. Following adequate weight restoration AB’s treatment plan as an OPD included psycho-education regarding the role of nutrition and exercise in health and variations in energy requirements for males during puberty and at different levels of activity. Identification of excessive or disordered exercise schedules were discussed along with examining the benefits of alternative and less intense exercise, such as mindfulness, yoga and walking. The prosocial aspect of exercise was also emphasised.

### Conclusion

Accepting that the clinical features of early onset AN may be atypical, it is important to maintain an open mind in cases presenting without goal directed weight loss. Although weight loss was significant in this case, its occurrence was due to an excessive drive to exercise without increasing calorific intake. In AB’s case, his exercise schedule might be seen as a coping mechanism to the ongoing stress and loss of structure brought about by Covid-19 restrictions. His failure to recognise bodily hunger signs and his general disinterest in food contributed to his weight loss. Maternal delayed awareness was associated with additional roles and stressors brought about by Covid-19, such as working from home, temporarily single-handedly parenting 5 children, concern for her husband quarantined abroad. The relatively short resolution of AB’s difficulties lay in the absence of pre-morbid psycho-pathology, the family’s structured yet nurturing style and the flexibility offered by the hospital and community services to adapt during a time of Covid-19 induced unprecedented challenges.

## Data Availability

Additional information available from the author upon request.

## Abbreviations

AB: Pseudonym initials given to boy
AN: Anorexia nervosa AN
BMI: Body mass Index
IBW: Ideal body weight
EDE-Q: Eating disorder examination questionnaire
CAMHS: Child and adolescent mental health services
ED: Eating disorder
EAI: Exercise addiction inventory
